# Synthesis of Gentiooligosaccharides of Genistein and Glycitein and Their Radical Scavenging and Anti-Allergic Activity

**DOI:** 10.3390/molecules16064740

**Published:** 2011-06-07

**Authors:** Kei Shimoda, Naoji Kubota, Hatsuyuki Hamada, Hiroki Hamada

**Affiliations:** 1 Department of Chemistry, Faculty of Medicine, Oita University, 1-1 Hasama-machi, Oita 879-5593, Japan; 2 National Institute of Fitness and Sports in Kanoya, 1 Shiromizu-cho, Kagoshima 891-2390, Japan; 3 Department of Life Science, Okayama University of Science, 1-1 Ridai-cho, Kita-ku, Okayama 700-0005, Japan

**Keywords:** gentiooligosaccharide, genistein, glycitein, DPPH free-radical scavenging activity, superoxide-radical scavenging activity, anti-allergic activity

## Abstract

The synthesis of gentiooligosaccharides of genistein and glycitein using cultured cells of *Eucalyptus perriniana* as biocatalysts was investigated. The cells of *E. perriniana* glycosylated genistein and glycitein to give the corresponding 4'-*O*-*β*-glucosides, 7-*O*-*β*-glucosides, and 7-*O*-*β*-gentiobiosides, which were two new compounds. The *β*-glucosides of genistein and glycitein showed 2,2-diphenyl-1-picrylhydrazyl (DPPH) free-radical scavenging activity and superoxide-radical scavenging activity. On the other hand, 7-*O*-*β*-glucosides of genistein and glycitein and the 7-*O*-*β*-gentiobioside of glycitein exerted inhibitory effects on IgE antibody production.

## 1. Introduction

The soy isoflavonoids genistein and glycitein have been recognized as natural antioxidants, and have been reported to show anti-allergic activities, such as inhibitory effects on histamine release from mast cells [1,2,3,4,5]. However, their use as food-ingredients is limited because of their water-insolubility and low absorbability after oral administration. Glycosylation using cultured cells is useful for preparing water-soluble and stable glycosides from water-insoluble and unstable organic compounds [6,7,8,9,10,11]. For example, it has been reported that glycosylation of the lipophilic flavonoid quercetin improved its absorbability after oral administration [12]. From the viewpoint of pharmacological development of isoflavonoids, their glycosylation is thus of importance. We report herein the biocatalytic glycosylation of genistein and glycitein by cultured cells of *Eucalyptus perriniana* to produce the corresponding 4'-O-*β*-glucosides, 7-O-*β*-glucosides, and 7-O-*β*-gentiobiosides. We also report their 2,2-diphenyl-1-picrylhydrazyl (DPPH) radical scavenging activity, superoxide-radical scavenging activity, and inhibitory activity for IgE antibody formation.

## 2. Results and Discussion

### 2.1. Glycosylation of Genistein *(**1**)* and Glycitein *(**5**)* by Cultured Cells of E. perriniana

**Figure 1:**
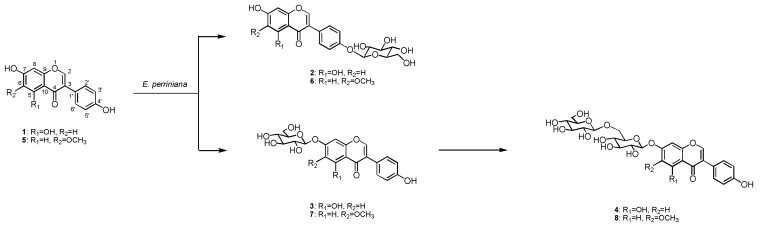
Synthesis of *β*-gentiooligosaccharides of genistein (**1**) and glycitein (**5**) by glycosylation with *E. perriniana.*

After cultured cells of *E. perriniana* were incubated with genistein (**1**) for five days, the glycosylated products **2**-**4** were isolated from the cells by extraction with MeOH. On the other hand, none were detected in the medium. No additional glycosylation products were detected in the MeOH extracts of the cells despite careful HPLC analyses. On the basis of their HRFABMS, ^1^H- and ^13^C-NMR, H-H COSY, C-H COSY, and NOE-spectroscopic analyses, the products (Figure 1) were determined to be genistein 4'-*O*-*β*-glucoside (**2**, 5%), genistein 7-*O*-*β*-glucoside (**3**, 41%), and genistein 7-*O*-*β*-gentiobioside (**4**, 3%), of which **4** is a new compound. 

The molecular formula of **4** was established as C_27_H_30_O_15_ based on its HRFABMS spectrum, which included a pseudomolecular ion [M + Na]^+^ peak at *m*/*z* 617.1488 (calcd. 617.1482 for C_27_H_30_O_15_Na). HRFABMS suggested that **4** was composed of one molecule of **1** and two hexoses. Its ^1^H-NMR spectrum showed two anomeric proton signals at *δ* 4.25 (1H, *d*, *J* = 8.0 Hz) and 5.10 (1H, *d*, *J* = 7.6 Hz). This suggested the presence of two *β*-anomers. The ^13^C-NMR spectrum included two anomeric carbon signals at *δ* 99.5 and 102.8. The sugar component of **4** was determined to be *β*-D-glucopyranose based on the chemical shifts of the carbon signals. The ^13^C resonance of C-6'' was shifted downfield to *δ* 68.6. Correlations were observed between the anomeric proton signal at *δ* 5.10 (H-1'') and the carbon signal at *δ* 163.0 (C-7), and between the anomeric proton signal at *δ* 4.25 (H-1''') and the carbon signal at *δ* 68.6 (C-6'') in the HMBC spectrum. These findings confirmed that the inner glucopyranosyl residue was attached to the phenolic hydroxyl group at C-7 of genistein (**1**), and that the pair of *β*-D-glucopyranosyl residues was 1,6-linked. Thus, **4** was identified as genistein 7-*O*-[6-O-(*β*-D-glucopyranosyl)]-*β*-D-glucopyranoside (7-*O*-*β*-gentiobioside).

Next, glycitein (**5**) was subjected to the same biotransformation system. Glycoside products **6**-**8** were obtained from the MeOH extracts of the cells. The products were identified as glycitein 4'-*O*-*β*-glucoside (**6**, 7%), glycitein 7-*O*-*β*-glucoside (**7**, 35%), and glycitein 7-*O*-*β*-gentiobioside (**8**, 2%) (Figure 1), of which **8** is new.

The HRFABMS spectrum of **8** included a pseudomolecular ion [M + Na]^+^ peak at *m*/*z* 631.1645, consistent with a molecular formula of C_28_H_32_O_15_ (calcd. 631.1639 for C_28_H_32_O_15_Na). The ^1^H-NMR spectrum of **8** included proton signals at *δ* 4.50 (1H, *d*, *J* = 7.6 Hz) and 5.12 (1H, *d*, *J* = 7.6 Hz), indicating the presence of two *β*-anomers in the sugar moiety. The ^1^H- and ^13^C-NMR spectra of **8** indicated that it was a *β*-gentiobiosyl analogue of **5**. Furthermore, the HMBC spectrum included correlations between the anomeric proton signal at *δ* 5.12 (H-1'') and the carbon signal at *δ* 152.5 (C-7), and between the anomeric proton signal at *δ* 4.50 (H-1''') and the carbon signal at *δ* 68.9 (C-6''). These findings confirm that the inner *β*-D-glucopyranosyl residue was attached to the phenolic hydroxyl group at C-7 of glycitein (**5**) and that the pair of *β*-D-glucopyranosyl residues were 1,6-linked. Thus, the product **8** was identified as glycitein 7-*O*-[6-O-(*β*-D-glucopyranosyl)]-*β*-D-glucopyranoside (7-*O*-*β*-gentiobioside).

### 2.2. Radical Scavenging Activity of β-Glycosides of Genistein and Glycitein

The DPPH free-radical scavenging activity of genistein *β*-glycosides **2**-**4** and glycitein *β*-glycosides **6**-**8** was determined by *in vitro* bioassay. The antioxidant activities were expressed as IC_50_ values and are summarized in Table 1. Genistein 4'-*O*-*β*-D-glucoside (**2**), genistein 7-*O*-β-D-glucoside (**3**), glycitein 4'-*O*-*β*-D-glucoside (**6**), and glycitein 7-*O*-*β*-D-glucoside (**7**) showed DPPH free-radical scavenging activity. On the other hand, the *β*-gentiobiosides of genistein and glycitein (**4** and **8**) had no antioxidant activity. The results obtained here suggested that mono-glucosides of genistein and glycitein would be useful free-radical scavenging antioxidants with aqueous-solubility.

The superoxide-radical scavenging activity of genistein *β*-glycosides **2**-**4** and glycitein *β*-glycosides **6**-**8** was expressed as IC_50_ values summarized in Table 1. Genistein 4'-*O*-*β*-D-glucoside (**2**), genistein 7-*O*-*β*-D-glucoside (**3**), glycitein 4'-*O*-*β*-D-glucoside (**6**), and glycitein 7-*O*-*β*-D-glucoside (**7**) showed superoxide-radical scavenging activity. The present data demonstrated that gentiobiosylation of isoflavones drastically reduced the antioxidant activity in comparison with isoflavone glucosides. A recent paper reported that glycitein 7-*O*-maltotrioside showed no antioxidant activity, although glycitein 7-*O*-glucoside exerted relatively high antioxidant activity, and that maltosylation at C-7 of glycitein also reduced the antioxidant activity in comparison with glycitein 7-*O*-glucoside [13].

These suggest that formation of long oligosaccharide chains attached to isoflavones might reduce their antioxidant activity. In addition, gentiobioside compounds which contains one phenolic hydroxyl group in their molecule, i.e., salicifolioside A, have been reported to show no DPPH free-radical scavenging activity [14]. It is postulated that gentiobiosylation might eliminate the antioxidant activity of aglycones. The results obtained here suggested that mono-glucosides of genistein and glycitein would be potential superoxide-radical scavenging antioxidants. Further studies on Trolox equivalent antioxidant capacity (TEAC) of the glycosides are now in progress.

**Table 1:** Antioxidant activities of genistein *β*-glycosides **2**-**4** and glycitein *β*-glycosides **6**-**8**.

Compound	IC_50_ (μM)	
	DPPH free-radical scavenging	Superoxide-radical scavenging
**2**	52	751
**3**	49	704
**4**	>200	>1000
**6**	55	764
**7**	51	708
**8**	162	>1000
**Vitamin C**	35	698

### 2.3. Anti-Allergic Activity of β-Glycosides of Genistein and Glycitein

The effects of genistein *β*-glycosides **2**-**4** on IgE antibody formation were examined by an *in vivo* bioassay using 7S-globulin from soybean as an antigen. The average of rat plasma IgE level after treatment of 7S-globulin with or without test compounds is summarized in Table 2. Genistein 7-*O*-*β*-D-glucoside (**3**) showed inhibitory action on IgE antibody generation. On the other hand, genistein 4'-*O*-*β*-D-glucoside (**2**) and genistein 7-*O*-*β*-gentiobioside (**4**) did not exhibit the inhibitory action on IgE antibody formation.

**Table 2:** Suppressive action of genistein and glycitein and their *β*-glycosides **1**-**8** on IgE antibody formation.

Compound	IgE level ^a^
**None**	401.7
**1**	145.1
**2**	452.2
**3**	168.5
**4**	466.2
**5**	135.9
**6**	383.8
**7**	151.0
**8**	256.6
**Hydrocortisone**	342.0

^a^ The results were expressed as average of plasma IgE level of seven rats administered a total of 10 mg/kg of each test compound.

Next, the average of rat plasma IgE level after treatment of 7S-globulin with or without glycitein *β*-glycosides **6**-**8** was examined. Glycitein 7-*O*-*β*-D-glucoside (**7**) and glycitein 7-*O*-*β*-gentiobioside (**8**) showed inhibitory action on IgE antibody formation. The 4'-*O*-*β*-D-glucoside (**6**) of glycitein did not inhibit IgE antibody generation (Table 2).

Recently, we reported that 7-*O*-*β*-glycosides of genistein and quercetin showed anti-allergic activity, *i.e.*, suppressive action on histamine release from rat peritoneal mast cells, whereas the *β*-glycosides, the sugar of which attached at other phenolic hydroxyl groups, exhibited no anti-allergic actions [15]. The results obtained here suggested that *β*-glucoside and *β*-gentiobioside at C-7 of genistein and/or glycitein did not attenuate the anti-allergic activity, and that phenolic hydroxyl groups at 4'-position might be necessary for the anti-allergic action of glycosides of genistein and glycitein. Further studies on the mechanism of *β*-glycosides of genistein and glycitein to act as anti-allergic formulations are now in progress.

## 3. Experimental Section

### 3.1. General

Genistein and glycotein were purchased from Sigma-Aldrich Co. The NMR spectra were recorded in DMSO-*d*_6_ using a Varian XL-400 spectrometer. The chemical shifts were expressed in *δ* (ppm) referring to tetramethylsilane. The HRFABMS spectra were measured using a JEOL MStation JMS-700 spectrometer. HPLC was carried out on a YMC-Pack R&D ODS column (150 × 30 mm) [solvent: CH_3_CN:H_2_O (3:17, v/v); detection: UV (280 nm); flow rate: 1.0 mL/min]. 

### 3.2. Cultured Cells and Culture Conditions

A cell culture of *E. perriniana* was induced in our laboratory, and has been cultivated for over 20 years [16]. Cultured *E. perriniana* cells were subcultured at 4-week intervals on solid Murashige and Skoog (MS) medium (100 mL in a 300-mL conical flask) containing 3% sucrose, 10 mM 2,4-dichlorophenoxyacetic acid, and 1% agar (adjusted to pH 5.7) at 25 °C in the dark. A suspension culture was started by transferring the cultured cells to 100 mL of liquid medium in a 300-mL conical flask, and incubated on a rotary shaker (120 rpm) at 25 °C in the dark. Prior to use for this work, part of the callus tissues (fr. wt 40 g) was transplanted to freshly prepared MS medium (100 mL in a 300-mL conical flask) and grown with continuous shaking for 2 weeks on a rotary shaker (120 rpm).

### 3.3. Production of β-Glycosides of Genistein and Glycitein by E. perriniana

Substrate (0.08 mmol) dissolved in EtOH (300 μL) was individually administered to a 500-mL flask containing suspension cultured cells of *E. perriniana*. The cultures were then incubated at 25 °C for five days on a rotary shaker (120 rpm) in the dark. After incubation, the cells and medium were separated by filtration with suction. The filtered medium was extracted with EtOAc. The medium was further extracted with *n*-BuOH. EtOAc and *n*-BuOH fractions were analyzed by HPLC The cells were extracted with MeOH for 12 h and sonicated for 5 min. The yields of the glycosylation products were calculated on the basis of the peak area from HPLC using calibration curves prepared by HPLC analyses of the authentic glycosides. The MeOH fraction was conc. and partitioned between H_2_O and EtOAc. The EtOAc fractions were combined and analyzed by the HPLC. The H_2_O fraction was applied to a Diaion HP-20 column and the column was washed with H_2_O followed by elution with MeOH. The MeOH eluate was subjected to HPLC [column: YMC-Pack R&D ODS column (150 × 30 mm); solvent: MeOH-H_2_O (9:11, v/v); detection: UV (280 nm); flow rate: 1.0 mL/min] to give glycosylated products.

Spectral data of new compounds are as follows.

*Genistein 7-O-β-gentiobioside* (**4**): IR (ν_max_): 3417, 1655, 1612, 1582, 1510, 1495, 1360, 1241; HRFABMS: *m*/*z* 617.1488 [M + Na]^+^ (calc. for C_27_H_30_O_15_Na, 617.1482); ^1^H-NMR: *δ* 3.15-3.70 (12H, m, H-2'', 2''', 3'', 3''', 4'', 4''', 5'', 5''', 6'', 6'''), 4.25 (1H, d, *J* = 8.0 Hz, H-1'''), 5.10 (1H, d, *J* = 7.6 Hz, H-1''), 6.51 (1H, d, *J* = 2.0 Hz, H-6), 6.70 (1H, d, *J* = 2.0 Hz, H-8), 6.90 (2H, d, *J* = 6.4 Hz, H-3', 5'), 7.51 (2H, d, *J* = 6.4 Hz, H-2', 6'), 8.40 (1H, s, H-2); ^13^C-NMR: *δ* 60.8 (C-6'''), 68.6 (C-6''), 69.2 (C-4'''), 69.8 (C-4''), 72.7 (C-2'''), 73.3 (C-2''), 75.9 (C-5'''), 76.3 (C-5''), 76.4 (C-3'''), 76.9 (C-3''), 99.5 (C-1''), 100.2 (C-8), 102.8 (C-1'''), 101.6 (C-6), 106.0 (C-10), 115.0 (C-3', C-5'), 122.1 (C-1'), 124.1 (C-3), 130.2 (C-2', C-6'), 153.5 (C-2), 156.2 (C-4'), 157.5 (C-9), 160.2 (C-5), 163.0 (C-7), 178.0 (C-4). Anal. Calcd for C_27_H_30_O_15_: C, 52.54; H, 4.90. Found: C, 52.45; H, 4.85.

*Glycitein 7-O-β-genitiobioside* (**8**): IR (ν_max_): 3410, 1651, 1611, 1577, 1510, 1490, 1362, 1235; HRFABMS *m/z*: 631.1645 [M + Na]^+^ (calcd 631.1639 for C_28_H_32_O_15_Na); ^1^H-NMR: *δ* 3.09-3.72 (12H, m, H-2'', 2''', 3'', 3''', 4'', 4''', 5'', 5''', 6'', 6'''), 3.90 (3H, s, OCH_3_), 4.50 (1H, d, *J* = 7.6 Hz, H-1'''), 5.12 (1H, d, *J* = 7.6 Hz, H-1''), 7.04 (2H, d, *J* = 8.0 Hz, H-3', 5'), 7.46 (1H, s, H-8), 7.51 (2H, d, *J* = 8.0 Hz, H-2', 6'), 7.76 (1H, s, H-5), 8.41 (1H, s, H-2); ^13^C-NMR: *δ* 56.1 (OCH_3_), 60.5 (C-6'''), 68.9 (C-6''), 69.5 (C-4'''), 69.9 (C-4''), 72.9 (C-2'''), 73.3 (C-2''), 75.7 (C-5'''), 76.7 (C-5''), 76.8 (C-3'''), 77.0 (C-3''), 99.0 (C-1''), 101.9 (C-1'''), 101.0 (C-8), 105.1 (C-5), 115.1 (C-3', C-5'), 117.6 (C-10), 122.5 (C-1'), 124.5 (C-3), 130.3 (C-2', C-6'), 148.8 (C-6), 151.0 (C-9), 152.5 (C-7), 153.5 (C-2), 157.0 (C-4'), 178.0 (C-4). Anal. Calcd for C_28_H_32_O_15_: C, 53.28; H, 5.11. Found: C, 53.25; H, 5.01.

### 3.4. DPPH Radical Scavenging Activity

DPPH free-radical scavenging activities of *β*-glycosides of genistein and glycotein were determined as follows: DPPH was dissolved in ethanol (500 μM) [17]. The sample solutions were prepared by dissolving each compound in ethanol. To solutions containing various concentrations of each sample (0.1 mL) and ethanol (0.9 mL) was added DPPH solution (1 mL) at room temperature. Vitamin C was used as a positive control. After 20 min at 25 °C, the absorbance was measured at 517 nm. The percentage reduction of the initial DPPH adsorption, i.e., the free-radical scavenging activity, was calculated as follows: E = [(*A*_c_ − *A_t_*) / *A*_c_] × 100, where *A*_t_ and *A*_c_ are the respective absorbance at 517 nm of sample solutions with and without the test compounds. Antioxidant activity was expressed as the 50% inhibitory concentration (IC_50_).

### 3.5. Superoxide-Radical Scavenging Activity

Superoxide was generated by the xanthine-xanthine oxidase system [17]. Reaction mixture contained 4 mM xanthine (50 μL), various concentration of sample in ethanol (50 μL), 2 mM nitro blue tetrazolium (NBT, 50 μL), 0.3 nkat/mL xanthine oxidase (50 μL), and 0.1 M phosphate buffer (pH 7.4) in a total volume of 2 mL. Vitamin C was used as a positive control. The reaction mixture was incubated at 25 °C for 10 min and the absorbance was read at 560 nm. Percent inhibition was calculated by comparing with control without test compound but containing the same amount of alcohol. The IC_50_ value is shown as the sample concentration at which 50% of superoxide-radical was scavenged.

### 3.6. Suppressive Action on IgE Antibody Formation

The inhibitory action of *β*-glycosides of genistein and glycotein on IgE antibody formation was examined as follows. 7S-Globulin was used as the antigen (1 mg/rat), and Al(OH)_3_ and pertussis vaccine were used as the adjuvants (20 mg and 0.6 mL/rat, respectively). Sensitization was made by injection of a mixture (0.6 mL) of the antigen and the adjuvant into the paws of each rat (male, ca. 200 g). Paw edema was measured 24 h after injection and the treated rats were divided in groups with an equal average swelling volume. Each sample was dissolved in physiological saline containing 10% Nikkol and the solution was injected daily into the rat for 11 d starting on the day of grouping. Hydrocortisone was used as the positive control. The amount of IgE was measured by the passive cutaneous anaphylaxis method on the 15th day [18]. The results were expressed as average of plasma IgE level of 7 rats administered a total of 10 mg/kg of each test compound.

## 4. Conclusions

The gentiooligosaccharides of genistein and glycitein were successfully produced by biocatalytic glycosylation with cultured cells of *E. perriniana*. The *β*-glucosides of genistein and glycitein showed DPPH free-radical scavenging activity and superoxide-radical scavenging activity. On the other hand, 7-*O*-*β*-glucosides of genistein and glycitein, and 7-*O*-*β*-gentiobioside of glycitein exerted inhibitory effects on IgE antibody production.

## References

[1] Adlercreutz H., Goldin B.R., Gorbach S.L., Höckerstedt K.A.V., Watanabe S. (1995). Soybean phytoestrogen intake and cancer risk. J. Nutr..

[2] Barnes S. (1998). Evolution of the health benefits of soy isoflavones. Proc. Soc. Exp. Biol. Med..

[3] Marotta F., Mao G.S., Liu T., Chui D.H., Lorenzetti A., Xiao Y., Marandola P. (2006). Anti-inflammatory and neuroprotective effect of a phytoestrogen compound on rat microglia. Ann. N.Y. Acad. Sci..

[4] Setchell K.D.R., Cassidy A. (1999). Dietary isoflavones: Biological effects and relevance to human health. J. Nutr..

[5] Chacko B.K., Chandler R.T., D’Alessandro T.L., Mundhekar A., Khoo N.K., Botting N., Barnes S., Patel R.P. (2007). Anti-inflammatory effects of isoflavones are dependent on flow and human endothelial cell PPARgamma. J. Nutr..

[6] Shimoda K., Kondo Y., Nishida T., Hamada H., Nakajima N., Hamada H. (2006). Biotransformation of thymol, carvacrol, and eugenol by cultured cells of *Eucalyptus perriniana*. Phytochemistry.

[7] Shimoda K., Kwon S., Utsuki A., Ohiwa S., Katsuragi H., Yonemoto N., Hamada H., Hamada H. (2007). Glycosylation of capsaicin and 8-nordihydrocapsaicin by cultured cells of *Catharanthus roseus*. Phytochemistry.

[8] Shimoda K., Kubota N., Kondo Y., Sato D., Hamada H. (2009). Glycosylation of fluorophenols by plant cell cultures. Int. J. Mol. Sci..

[9] Shimoda K., Akagi M., Hamada H. (2009). Production of *β*-maltooligosaccharides of *α*- and *δ*-tocopherols by *Klebsiella pneumoniae* and cyclodextrin glucanotransferase as anti-allergic agents. Molecules.

[10] Shimoda K., Hamada H. (2010). Production of hesperetin glycosides by *Xanthomonas campestris* and cyclodextrin glucanotransferase and their anti-allergic activities. Nutrients.

[11] Shimoda K., Kubota N., Taniuchi K., Sato D., Nakajima N., Hamada H., Hamada H. (2010). Biotransformation of naringin and naringenin by cultured *Eucalyptus perriniana* cells. Phytochemistry.

[12] Morand C., Manach C., Crespy V., Remesy C. (2000). Quercetin 3-*O*-beta-glucoside is better absorbed than other quercetin forms and is not present in rat plasma. Free Radic. Res..

[13] Shimoda K., Hamada H. (2010). Synthesis of *β*-maltooligosaccharides of glycitein and daidzein and their anti-oxidant and anti-allergic activities. Molecules.

[14] Calis I., Kuruuzum A., Demirezer O., Sticher O., Ganci W., Ruedi P. (1999). Phenyl valeric acid and flavonoid glycosides from *Polygonum salicifolium*. J. Nat. Prod..

[15] Shimoda K., Kobayashi T., Akagi M., Hamada H., Hamada H. (2008). Synthesis of oligosaccharides of genistein and quercetin as potential anti-inflammatory agents. Chem. Lett..

[16] Furuya T., Orihara Y., Hayashi C. (1987). Triterpenoids from *Eucalyptus perriniana* cultured cells. Phytochemistry.

[17] Ozsoy N., Candoken E., Akev N. (2009). Implications for degenerative disorders: Antioxidative activity, total phenols, flavonoids, ascorbic acid, *β*-carotene and *β*-tocopherol in *Aloe vera*. Oxid. Med. Cell Longev..

[18] Koda A., Miura T., Inagaki N., Sakamoto O., Arimura A., Nagai H., Mori H. (1990). A method for evaluating anti-allergic drugs by simultaneously induced passive cutaneous anaphylaxis and mediator cutaneous reactions. Int. Arch. Allergy. Appl. Immunol..

